# Transplantation of human cord blood mononuclear cells and umbilical cord-derived mesenchymal stem cells in autism

**DOI:** 10.1186/1479-5876-11-196

**Published:** 2013-08-27

**Authors:** Yong-Tao Lv, Yun Zhang, Min Liu, Jia-na-ti Qiuwaxi, Paul Ashwood, Sungho Charles Cho, Ying Huan, Ru-Cun Ge, Xing-Wang Chen, Zhao-Jing Wang, Byung-Jo Kim, Xiang Hu

**Affiliations:** 1 Shandong Jiaotong Hospital, Jinan, Shandong, China; 2 Shenzhen Beike Cell Engineering Research Institute, 2/F, Yuanxing Technology Building, #1 Songpingshan Street, Nanshan District, Shenzhen, Guangdong 518057, China; 3 Department of Medical Microbiology & Immunology, University of California Davis, Davis, CA, USA; 4 Department of Neurology and Neurosurgery, Stanford University, Stanford, CA, USA; 5 Department of Neurology, Korea University Anam Medical Center, Seoul, Korea

**Keywords:** Autism, Cord blood mononuclear cell, Umbilical cord-derived mesenchymal stem cell, Cell transplantation

## Abstract

**Background:**

Autism is a pervasive neurodevelopmental disorder. At present there are no defined mechanisms of pathogenesis and therapy is mostly limited to behavioral interventions. Stem cell transplantation may offer a unique treatment strategy for autism due to immune and neural dysregulation observed in this disease. This non-randomized, open-label, single center phase I/II trial investigated the safety and efficacy of combined transplantation of human cord blood mononuclear cells (CBMNCs) and umbilical cord-derived mesenchymal stem cells (UCMSCs) in treating children with autism.

**Methods:**

37 subjects diagnosed with autism were enrolled into this study and divided into three groups: CBMNC group (14 subjects, received CBMNC transplantation and rehabilitation therapy), Combination group (9 subjects, received both CBMNC and UCMSC transplantation and rehabilitation therapy), and Control group (14 subjects, received only rehabilitation therapy). Transplantations included four stem cell infusions through intravenous and intrathecal injections once a week. Treatment safety was evaluated with laboratory examinations and clinical assessment of adverse effects. The Childhood Autism Rating Scale (CARS), Clinical Global Impression (CGI) scale and Aberrant Behavior Checklist (ABC) were adopted to assess the therapeutic efficacy at baseline (pre-treatment) and following treatment.

**Results:**

There were no significant safety issues related to the treatment and no observed severe adverse effects. Statistically significant differences were shown on CARS, ABC scores and CGI evaluation in the two treatment groups compared to the control at 24 weeks post-treatment (p < 0.05).

**Conclusions:**

Transplantation of CBMNCs demonstrated efficacy compared to the control group; however, the combination of CBMNCs and UCMSCs showed larger therapeutic effects than the CBMNC transplantation alone. There were no safety issues noted during infusion and the whole monitoring period.

**Trial registration:**

ClinicalTrials.gov: NCT01343511, Title “Safety and Efficacy of Stem Cell Therapy in Patients with Autism”.

## Introduction

Autism spectrum disorders (ASD) are heterogeneous neurodevelopmental disorders [1]. Autism is the most prevalent ASD, characterized by dysfunctions in reciprocal social interaction and communication, as well as presence of repetitive and stereotypical behaviors [2]. Recent reports have highlighted the dramatic rise in the number of children affected with autism (57% growth compared with 2002) with current prevalence rate in the USA approaching 1% [3]. The exact etiology of autism remains unclear. Research data have revealed that autism may result from a complex combination of genetic and environmental factors [2,4] and is associated with several biochemical events, including ongoing cerebral hypoperfusion; immune dysregulation and activation of neuroglial cells; decreased methylation capacity; limited production of glutathione; mitochondrial dysfunction; intestinal dysbiosis and oxidative stress [5]. In addition, the triggering pathophysiology and subsequent mechanisms of pathogenesis have not been clarified. Consequently, identifying effective treatments for autism is particularly difficult.

Current available therapeutic approaches for autism can be broadly classified into behavioral, educational, medical, allied health, and complementary and alternative medicine interventions [6]. However, there is no defined standard treatment intervention for children with autism [7,8]. Recently, stem cell therapy showed great promise for the future of regenerative medicines. Pre-clinical studies reported that human cord blood mononuclear cell (CBMNC) transplantation in animal models of brain ischemia could promote functional recovery by improving local blood perfusion to damaged areas through angiogenesis [9,10]. In addition, the CBMNC was safely transplanted for clinical applications in non-hematopoietic degenerative conditions in the absence of immune suppression [11,12]. Other data revealed that mesenchymal stem cells (MSCs) represent remarkable immunoregulatory properties by suppressing proliferation and function of several major immune cells, including T cells, B cells, natural killer (NK) cells, modulating the activities of dendritic cells (DC) and inducing regulatory T cells [13,14]. The authors hypothesized that the combination of therapy modalities reveals robust preclinical and human safety evidence to allow further study in treating autism. This was a non-randomized, open-label, controlled, single-center phase I/II clinical trial to examine the treatment safety and efficacy of transplantation of CBMNCs and/or human umbilical cord-derived mesenchymal stem cells (UCMSCs) in children with autism.

## Methods

### Study population and design

The study protocol and Informed Consent Forms were approved by the Institutional Review Board of the Shandong Jiaotong Hospital under the auspices of the National Ministry of Health. Subjects were recruited from the Shandong Jiaotong Hospital and Shandong Rehabilitation Therapy Center between March and September 2009. Eligible subjects included male or female (3 ~ 12 years of age): diagnosed with autism, in accordance with the diagnostic criteria for autism in Diagnostic and Statistical Manual of Mental Disorders, Fourth Edition (DSM-IV) [15], and a score of Childhood Autism Rating Scale (CARS) ≥ 30. Exclusion criteria included: 1) prior history of severe allergic reactions; 2) any severe psychiatric disorder or an alternative ASD such as Asperger syndrome, Rett syndrome or undefined pervasive developmental disorders; 3) seizures within the past six months; 4) autism caused by active epilepsy, cerebrovascular diseases or brain trauma; 5) Severity of Illness (SI) of Clinical Global Impression (CGI) scale evaluated as “Normal” or “Borderline mentally ill” or “Mildly ill”; 6) moderate or severe extrapyramidal symptoms or tardive dyskinesia; 7) severe self-injury behavior; 8) active systemic or severe focal infections, including human immunodeficiency virus (HIV), syphilis and hepatitis; 9) autoimmune diseases; 10) severe pulmonary and hematological diseases, malignancy or hypoimmunity; 11) undertaking other treatments that may affect the safety and efficacy evaluation of stem cell therapy; 12) enrollment in other clinical trials in the last three months; 13) other clinical conditions that the investigators considered not appropriate for enrollment in this study.

**Figure 1:**
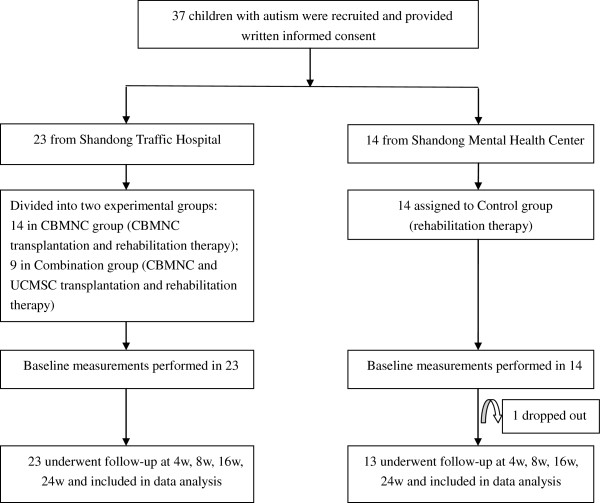
Enrollment and retention algorithm.

After getting the signed Informed Consent Form from individual subjects’ guardians, qualified investigators evaluated each subject’s condition based on the designed study protocol. Enrolled subjects were divided into three groups: the Control group, which received only rehabilitation therapy; the CBMNC group, which received CBMNC transplantation and rehabilitation therapy; and the Combination group, which received combined CBMNC and UCMSC transplantation and rehabilitation therapy. All subjects received the same professional sensory integration and behavioral rehabilitation therapy at Shandong Rehabilitation Therapy Center and had no other treatments except those prescribed as part of the trial during the study period. Each subject completed a 24-week follow-up period by June 2010 (Figure 1).

### Cell preparation and administration

The CBMNC and UCMSC were provided by *Shenzhen Beike Biotechnology Co., Ltd*. Fresh human cord blood and umbilical cord were obtained from informed healthy donors in accordance with the sterile procurement guidelines established by the hospital in conjunction with the National Ministry of Health. After collection, each sample was tested for communicable diseases, including hepatitis B, hepatitis C, HIV, cytomegalovirus and syphilis, as well as the enzyme of alanine aminotransferase, and then transferred for cell preparation in the GMP laboratories.

Cord blood was diluted with saline in the ratio 2:1 and 30 mls of the diluted blood was then added to 15 mls of Ficoll in every 50 ml centrifuge tube and then centrifuged (750 g × 22 minutes). Mononuclear cells were collected and washed twice in saline. Contaminating erythrocytes were lyzed with lysis buffer comprising of injection grade water. Cell density was adjusted to 2~6 × 10^6^/ml and seeded in Dulbecco’s modified Eagle’s medium: nutrient mixture F-12 culture medium with basic fibroblast growth factor and epidermal growth factor at a concentration of 20 ng/ml. Culture media was mixed with 2% v/v B-27 Stem Cell Culture Supplements. Cells were cultured at 37°C with saturated humidity and 5% CO2 by volume and harvested for clinical application after 4 ~ 7 days of cultivation. The final CBMNC product contains 0.2 ~ 1.0% CD34^+^ cells as determined by flow cytometry.

The umbilical cord was rinsed twice in normal saline and the cord blood was removed during this process. The washed cord was cut into 2-3 cm pieces and then bluntly dissected to obtain Wharton’s jelly. The Wharton’s jelly was cut into 1 ~ 4 mm^3^ pieces, floated in a flask with low-glucose Dulbecco’s modified Eagle’s medium containing serum substitute supplement, and subsequently incubated at 37°C in a humidified atmosphere consisting of 5% CO2. The medium was replaced every 3 ~ 5 days after the initial plating. When well-developed colonies of fibroblast-like cells appeared after 12 ~ 14 days, the cells were trypsinized and passaged into a new flask for further expansion. The UCMSCs harvested from passage 2 were used in this study and flow cytometry results showed that ≥95% of cells expressed CD29, CD73, CD90 and CD105, while the expression of CD45, CD34, CD14, CD79 and HLA-DR was 2% or less. The capacity of UCMSCs that differentiate into adipogenic and osteogenic lineages was identified and the soft agar cloning assay and tumorigenicity experiments with UCMSCs in mice showed no carcinogenicity.

To ensure the quality of CBMNCs and UCMSCs, cell growth was regularly monitored during the cultivation and all the inspection information was recorded accordingly, including test results for sterility, mycoplasma and endotoxin (≤0.5 EU/ml). Any contaminated cell suspensions or unhealthy cells were eliminated upon discovery. The finished cell product incorporated a final cell count as requested, cell viability (≥85%) determined by trypan blue testing and sterility test.

After extensive discussion answering all questions, written informed consents were obtained from each subject’s guardian before initiating the scheduled treatments. With the support from accumulated clinical experience of our exploratory stem cell treatments [11,12,16], the subjects recruited in the CBMNC and Combination groups received four cell transplantations at an interval of 5 ~ 7 days. Approximately 2 × 10^6^/kg CBMNCs and 1 × 10^6^/kg UCMSCs were infused with normal saline intravenously (20 ml) and/or intrathecally (2 ml), respectively, per treatment. The CBMNC group received the first transplantation through intravenous infusion and subsequent three transplantations through intrathecal injections. The Combination group received two CBMNC intravenous and intrathecal infusions each, followed by two UCMSC intrathecal injections.

### Safety and efficacy measure

Treatment safety was evaluated with: documentation of physical examination, vital signs and adverse events; complete blood count, liver and renal function, serum glucose, lipid profile, immunology testing (including immunoglobulin (Ig) A/G/M, complement C3/C4 and T-cell subsets) at baseline (pre-treatment) and 4 (4w), 8 (8w), and 24 (24w) weeks after the first cell transplantation. Subjects were assessed with CARS, CGI scale, and Aberrant Behavior Checklist (ABC) at baseline and 4w, 8w, 16w and 24w after the first cell transplantation for efficacy. All assessments were conducted by physicians from the Shandong Mental Health Center. The CARS and CGI evaluations were the predetermined primary outcome measures and the ABC was the secondary outcome measure.

CARS assesses behavior in 14 domains that are generally affected in autism, plus one general category for impressions of features of autism, with the aim to identify children with autism from other developmental disorders [17]. The total score was classified as “no autism (below 30)”, “mild or moderately autism (30 ~ 36.5)” or “severe autism (above 36.5)”. The CGI assessment, a widely used assessment tool in psychiatry for clinical trials, is a 3-item observer-rated scale that measures illness severity (SI), global improvement (GI) and therapeutic effects (EI) [18]. The SI is rated on a 7-point scale, ranging from 1 (normal) to 7 (most severely ill subjects) and GI scores range from 1 (very much improved) to 7 (very much worse). EI ratings take account of both therapeutic efficacy and treatment-related adverse events and range from 0 (marked improvement with no side-effects) and 4 (unchanged or worse with side-effects outweighing the therapeutic effects). Five subscales are designed in ABC to measure symptoms in children with autism, including “Irritability”, “Lethargy/Social Withdrawal”, “Stereotypic Behavior”, “Hyperactivity” and “Inappropriate Speech” and consisting of 58 items, each rated on a 4-point scale [19,20].

### Statistical analysis

Testing and evaluations were standardized and applied equally across all subjects. Quantitative Data were presented as means ± standard deviations (±s) and percentages were used to describe the qualitative data. Independent-samples t-test and Wilcoxon signed-rank test were used to compare the vital signs, including blood pressure and heart rate, at each follow-up to baseline measurements. Safety parameters were analyzed with chi-square test or Fisher’s exact test at pre- and post-treatment time points. To estimate the treatment effect, differences in CARS and ABC scores in each group were compared with multivariate analysis of variance (MANOVA) and in each time point using repeated measures analysis of variance (ANOVA). Qualitative data from CGI scales were analyzed using K independent samples rank sum test or two independent samples rank sum test. Pearson’s correlation coefficient was applied to correlate CARS and ABC scores. All statistical analyses were performed using SPSS 13.0 statistical package (SPSS, Chicago, IL, USA). A p-value <0.05 was considered statistically significant and all statistical tests were two-sided.

## Results

### Participant characteristics

A total of 37 participants with confirmed autism gave written informed consent to participate in this trial (Figure 1). Of these participants, 14 males from Shandong Rehabilitation Therapy Center were enrolled in the Control group, ranging in age from 3.51-10.02 (mean, 5.60 ± 2.01). One of the 14 control subjects dropped out for reasons unrelated to the trial and that dataset was not included in the safety and efficacy analyses. The remaining 23 subjects from Shandong Jiaotong Hospital were randomized to receive stem cell therapy: 14 subjects in the CBMNC group, including 13 males and 1 female, age ranging from 3.29-12.02 (mean, 7.41 ± 2.63), and 9 male subjects in the Combination group, ranging in age from 3.98-9.83 (mean, 6.20 ± 2.12). There were no statistically significant differences in the demographic data, including age, height and weight, among the three groups (P > 0.05, Table 1).

**Table 1:** Baseline characteristics of enrolled patients

**Variable**		**CBMNC**	**Combination**	**Control**
		**(N = 14)**	**(N = 9)**	**(N = 14)**
Gender	Male N(%)	13(92.86%)	9(100%)	14(100%)
	Female N(%)	1(7.14%)	0(0.00%)	0(0.00%)
Age (year)	Mean	7.41 ± 2.63	6.20 ± 2.12	5.60 ± 2.01
	Median	7.08	6.51	5.02
	Min-Max	3.29-12.02	3.98-9.83	3.51-10.02
Height(cm)	Mean	102.86 ± 19.29	98.89 ± 23.56	86.21 ± 12.07
	Median	105.00	90.00	82.50
	Min-Max	80.00-130.00	75.00-130.00	70.00-120.00
Weight(Kg)	Mean	28.32 ± 8.39	27.89 ± 7.85	21..5651 ± 7
	Median	29.25	26.00	19.50
	Min-Max	15.50-40.00	20.00-40.00	15.00-40.00

### Safety measurement

All subjects had normal and stable vital signs during the treatment course. Five subjects transiently developed low-grade fever (3 subjects in the CBMNC group and 2 in the Combination group) and recovered without medical interventions. No allergic, immunological reactions or other serious adverse events were observed at the time of injection or during the whole follow-up period in the two groups receiving stem cell transplantation. There were no deviations outside of reference ranges or significant liver/renal laboratory changes post-treatment compared to baseline (Table 2). Other safety measures, including complete blood count, serum glucose, lipid profile and immunological parameters (Ig A/G/M, C3/C4 and T-cell subsets), showed no significant changes from baseline.

**Table 2:** Serum biochemistry at pre- and post-treatment in the CBMNC and combination groups

**Parameter**	**ALT (U/L)**	**AST (U/L)**	**TBI (μmol/L)**	**BUN (μmol/L)**	**SCR (μmol/L)**
Reference range	0 ~ 40	0 ~ 50	1.7 ~ 20.5	3.2 ~ 7.1	44 ~ 133
Baseline	27.71 ± 9.82	34.67 ± 8.49	13.10 ± 7.20	5.67 ± 1.32	44.30 ± 8.20
4w	26.70 ± 9.42	35.00 ± 7.34	12.78 ± 4.33	6.10 ± 1.24	42.56 ± 7.46
8w	26.45 ± 8.56	35.12 ± 6.38	12.56 ± 4.21	6.21 ± 1.32	42.60 ± 7.66
24w	27.56 ± 8.46	35.76 ± 9.73	13,45 ± 6.15	5.72 ± 1.56	43.57 ± 8.43

Note: *ALT* denotes alanine aminotransferase, *AST* aspartate aminotransferase, *TBI* total bilirubin, *BUN* blood urea nitrogen, and *SCR* Serum creatinine.

### Primary outcomes

#### Childhood autism rating scale

Behavioral therapy alone in the Control group was effective at reducing total scores in CARS after 24w (Table 3). The total scores obtained following CARS assessment were decreased from 45.11 ± 4.31 at baseline to 28.00 ± 6.18 at 24w in the Combination group, 46.43 ± 8.65 at baseline to 37.14 ± 10.15 at 24w in the CBMNC group, and 43.15 ± 4.38 at baseline to 37.23 ± 3.42 at 24w in the Control group. Total CARS scores were significantly decreased in the Combination group compared with CBMNC and Control groups at 24w (Table 3; p < 0.05). The changes in total CARS scores represented a decrease of 37.9% in the Combination group, which were significantly different to the changes of 20.0% observed in the CBMNC group and 13.7% in the Control groups (Table 3, p < 0.05). In addition, there were significant differences for CARS scores in the CBMNC group at 4w, 8w and 16w compared with baseline (p < 0.05). The difference in CARS sub-scales were compared among the groups at each designed time point. Improvements in behaviors as determined for the sub-scales of “Relating to people”, “Body use“, “Visual response”, “Taste, smell, and touch response and use” and “General impressions” in the Combination group were observed when compared with the CBMNC and Control groups (p < 0.05) (see Additional file 1: Table S1, “CARS Scores”).

**Table 3:** CARS total score

**Group**	**Baseline**	**4w**	**8w**	**16w**	**24w**
CBMNC	46.43 ± 8.65	^*^39.21 ± 8.63	^*^36.64 ± 7.07	^*^35.14 ± 7.77	37.14 ± 10.15
Combination	45.11 ± 4.31	40.67 ± 3.82	38.22 ± 9.74	36.78 ± 12.8	^*^28.00 ± 6.18^ab^
Control	43.15 ± 4.38	41.54 ± 3.82	41.46 ± 3.41	40.31 ± 3.82	^*^37.23 ± 3.42

Note: ^*^Change in CARS scores at follow-up points compared to baseline in each group (p < 0.05). ^a^The CARS score of the Combination or CBMNC group at each evaluation point compared with the Control group (p < 0.05). ^b^The CARS score of the Combination group at each evaluation point compared with the CBMNC group (p < 0.05).

### Clinical global impression scale

At baseline, no significant differences among the three groups were observed in the CGI-SI scale (Table 4; p > 0.05). For the Control group 50% were scored as “Moderately ill”, 21% as “Markedly ill” and 21% as “Severely ill” at baseline (Table 4). However, there were statistically significant differences in CGI-SI in the Combination group compared with the Control group at 24w (Table 4; P < 0.05). At 24w, 92% of controls were considered “Moderately ill”, whereas in the Combination group, the assessments had improved with 11% considered “normal, not ill”, 55% “Mildly ill” and only 33% classified as “Markedly ill”. In comparison in the CBMNC group, 7% were considered “borderline”, 29% “Mildly ill”, 29% “Moderately ill”, 14% “Markedly ill” and 14% “Severely ill” at 24w. The frequency of participants that were “Very much improved” and “Much improved” based on the CGI-GI scale was increased in the Combination group (88.89%) and CBMNC group (50%) and was significantly different from the Control group (7.69%) at 24w (Table 5; p < 0.05). The frequency of participants of “Marked” and “Moderate” effects in the CGI-EI scale was also more pronounced in the Combination group (88.89%), and CBMNC group (50%) compared with the Control group (7.69%) at 24 W (Table 5; p < 0.05).

**Table 4:** CGI-SI scale at baseline and 24w

**Scale**	**Baseline**			**24w**		
	**Combination**	**CBMNC**	**Control**	^*^Combination^**ab**^	^*^CBMNC^**a**^	**Control**
0 - n(%)	0(0.00%)	0(0.00%)	0(0.00%)	0(0.00%)	0(0.00%)	1(7.69%)
1 - n(%)	0(0.00%)	0(0.00%)	0(0.00%)	1(11.11%)	0(0.00%)	0(0.00%)
2 - n(%)	0(0.00%)	0(0.00%)	0(0.00%)	0(0.00%)	1(7.14%)	0(0.00%)
3 - n(%)	2(22.22%)	1(7.14%)	1(7.14%)	5(55.56%)	4(28.57%)	0(0.00%)
4 - n(%)	2(22.22%)	5(35.71%)	7(50.00%)	3(33.33%)	4(28.57%)	12(92.31%)
5 - n(%)	2(22.22%)	1(7.14%)	3(21.43%)	0(0.00%)	2(14.29%)	0(0.00%)
6 - n(%)	3(33.33%)	6(42.86%)	3(21.43%)	0(0.00%)	2(14.29%)	1(7.69%)
7 - n(%)	0(0.00%)	1(7.14%)	0(0.00%)	0(0.00%)	1(7.14%)	0(0.00%)
Total (missed)	9(0)	14(0)	14(0)	9(0)	14(0)	13(1)

Note: 0 = Not assessed; 1 = Normal, not at all ill; 2 = Borderline mentally ill; 3 = Mildly ill; 4 = Moderately ill; 5 = Markedly ill; 6 = Severely ill; 7 = Among the most extremely ill patients. ^*^The frequency of participants who scored “Mildly ill” or better was significantly increased at 24w when compared to baseline (p < 0.05). ^a^The frequency of participants who scored “Mildly ill” or better in the Combination or CBMNC group was significantly increased when compared with the Control group at 24w (p < 0.05). ^b^The frequency of participants who scored “Mildly ill” or better in the Combination group was significantly increased when compared with the CBMNC group at 24w (p < 0.05).

**Table 5:** CGI-GI and CGI-EI scales at 24w

	** CGI scale**	**Combination**^**ab**^	**CBMNC**^**a**^	**Control**
CGI-GI	Not assessed	0(0.00%)	0(0.00%)	1(7.69%)
N (%)	Very much improved	3(33.33%)	1(7.14%)	0(0.00%)
	Much improved	5(55.56%)	6(42.86%)	1(7.69%)
	Minimally improved	1(11.11%)	2(14.29%)	11(84.62%)
	No change	0(0.00%)	5(35.71%)	1(7.69%)
	Minimally worse	0(0.00%)	0(0.00%)	0(0.00%)
	Much worse	0(0.00%)	0(0.00%)	0(0.00%)
	Very much worse	0(0.00%)	0(0.00%)	0(0.00%)
CGI-EI	Unchanged or worse	0(0.00%)	5(35.71%)	1(7.69%)
N (%)	Minimal	1(11.11%)	2(14.29%)	11(84.62%)
	Moderate	6(66.67%)	7(50.00%)	1(7.69%)
	Marked	2(22.22%)	0(0.00%)	0(0.00%)
	Total(missed)	9(0)	14(0)	13(1)

Note: ^a^The percent of “Very much improved” and “Much improved” in the CGI-GI scale and the percent of “Marked” and “Moderate” effects in the CGI-EI scale were significantly higher in the Combination and CBMNC groups when compared with the Control group at 24w (p < 0.05). ^b^The percent of “Very much improved” and “Much improved” in the CGI-GI scale and the percent of “Marked” and “Moderate” effects in the CGI-EI scale were significantly higher in the Combination group when compared with the CBMNC group at 24w (p < 0.05).

### Secondary outcomes

#### Aberrant behavior checklist

Compared with baseline assessments, there were significant decreases in total ABC scores at 24w in the Combination (59.9%), CBMNC group (38.0%) and the Control group (17.4%) (Table 6; p < 0.05). There were also significant differences in total ABC scores at 8w and 16w in the CBMNC group compared with baseline. At 24w post treatment, there were significant differences of “Lethargy/Social withdrawal”, “Stereotypic behavior” and total ABC scores in the Combination group when compared with the CBMNC and Control groups (p < 0.05). The scores were no statistical differences of “Hyperactivity”, “Irritability” or “Inappropriate speech” between the three groups at 24w (p > 0.05). Interestingly, there was close correlation between the assessment results of ABC and CARS at each evaluation point with strong associations between mean total scores of ABC and CARS at each follow-up point post treatment (see Additional file 1: Table S2, “Correlation of ABC and CARS Scores”; p < 0.001).

**Table 6:** ABC score

**ABC item**	**Group**	**Baseline**	**4w**	**8w**	**16w**	**24w**
Irritability	CBMNC	16.36 ± 9.61	14.00 ± 8.66	9.50 ± 8.81	10.21 ± 9.58	8.14 ± 8.37
	Combination	15.00 ± 7.81	11.22 ± 5.76	9.78 ± 4.32	5.67 ± 3.24	4.22 ± 3.19
	Control	9.15 ± 5.58	8.31 ± 5.41	8.46 ± 5.41	8.69 ± 5.56	6.92 ± 4.96
Lethargy/Social withdrawal	CBMNC	30.71 ± 6.08	26.86 ± 5.26^a^	24.07 ± 7.08^a^	23.86 ± 8.62^a^	24.14 ± 9.65^a^
	Combination	31.00 ± 6.98	25.78 ± 6.50^a^	22.67 ± 5.27^a^	21.11 ± 5.82^a^	^*^16.00 ± 7.92^ab^
	Control	35.08 ± 4.96	34.31 ± 5.14	33.92 ± 4.73	32.46 ± 5.55	^*^30.54 ± 5.03
Stereotypic behavior	CBMNC	29.43 ± 9.77	^*^22.43 ± 8.98	^*^18.86 ± 10.52	^*^18.00 ± 10.86	^*^17.07 ± 9.93
	Combination	28.33 ± 8.47	20.78 ± 8.81	17.22 ± 7.12	13.22 ± 6.78	^*^9.33 ± 5.81^ab^
	Control	22.77 ± 6.86	20.85 ± 6.63	21.08 ± 4.82	18.38 ± 5.58	17.31 ± 4.05
Hyperactivity	CBMNC	11.86 ± 4.55^a^	10.07 ± 4.63	7.57 ± 4.83	7.29 ± 5.27	6.86 ± 5.26
	Combination	11.89 ± 6.88^a^	9.33 ± 5.87	7.22 ± 5.19	5.89 ± 3.37	4.67 ± 3.74
	Control	6.08 ± 3.15	6.31 ± 2.81	6.46 ± 3.38	6.15 ± 3.39	5.08 ± 2.40
Inappropriate speech	CBMNC	5.71 ± 4.30^a^	3.79 ± 3.29	3.43 ± 3.34	2.93 ± 2.87	2.14 ± 2.32
	Combination	5.56 ± 2.83^a^	4.56 ± 2.92	3.11 ± 1.62	3.22 ± 2.44	2.56 ± 2.19
	Control	2.38 ± 2.10	2.69 ± 2.21	3.00 ± 2.27	2.92 ± 2.56	2.46 ± 2.63
Total score	CBMNC	94.07 ± 21.98	77.14 ± 22.4	^*^63.43 ± 29.93	^*^62.29 ± 33.98	^*^58.36 ± 31.73^a^
	Combination	91.78 ± 25.92	71.67 ± 22.57	60.00 ± 18.94	49.11 ± 14.61	^*^36.78 ± 16.95^ab^
	Control	75.46 ± 12.05	72.46 ± 12.95	72.92 ± 11.43	68.62 ± 12.58	^*^62.31 ± 11.3

Note: ^*^Aberrant behavioral scores at different follow-up time-points compared with baseline scores in each group (p < 0.05). ^a^Aberrant behavioral scores of the Combination or CBMNC group compared with the Control group (p < 0.05). ^b^Comparison of aberrant behavioral scores of the Combination group with the CBMNC group (p < 0.05).

## Discussion

In the current proof-of-concept study we used CBMNC and UCMSC transplantation in addition to conventional behavioral therapies to test the potential effects of stem cell therapies in children with autism. The preliminary data confirms the CBMNC and UCMSC transplantation was safe and efficacious at the doses, formulation, method of delivery and intervals treated. Compared with the Control group, both objective functional and subjective improvements were observed in visual, emotional and intellectual responses, body use, adaption to change, fear or nervousness, nonverbal communication and activity level assessed by CARS, as well as in lethargy/social withdrawal, stereotypic behavior, hyperactivity and inappropriate speech evaluated by ABC in both the CBMNC and Combination groups. Similarly, the CGI-SI, CGI-GI and CGI-EL also showed statistical significant improvements when compared with the Control group. Safety measurements indicated that stem cell administration via intravenous and intrathecal infusions were well tolerated without immediate or long term side effects during the 24-week follow-up period. The few cases with low-grade fever were mild and resolved without special medical interventions. With these results in this small cohort of subjects, the risk-benefit ratio of stem cell therapy in autistic children appears to be favorable.

While the pathophysiology of autism remains poorly defined, accumulating data suggests that one potential etiology may involve immune dysregulation (reviewed in Onore et al. 2012) [21]. Extensive data indicate an abnormal immune system, including active neuroinflammation in the brain, elevated pro-inflammatory cytokine profiles, dysfunction of immune cells and presence of autoimmunity, directly related to increased impairments in behavior. Studies demonstrated an ongoing neuroinflammatory process with marked activation of microglia and astroglia in the cerebral cortex, white matter and cerebellum of individuals with autism [22]. A unique proinflammatory cytokine profile in autistic patients has been documented in the cerebrospinal fluid, including a marked increase in macrophage chemoattractant protein-1 [22], and in the peripheral plasma, such as significantly elevated levels of interleukin (IL)-1β, IL-8 and IL-12p40 [23]. Additional studies found altered function in immune cell subsets [24-26], leading to an inappropriate or ineffective immune response to pathogen challenge in autism. Various autoantibodies responding to the proteins in central nervous system have been detected in the children with autism, which might link autism with an autoimmune process rather than an externally triggered immune reaction [27-29]. Collectively, these data suggest that immune dysfunction is not only a symptom/co-morbidity but indicative of an underlying pathophysiological process, so that targeting this pathology and modifying neuroimmune reactions may be productive from the therapeutic perspective. However, few clinical trials of anti-inflammatory drugs have aimed to correct immune dysregulation/ongoing neuroinflammation in autism. Due to their known ability to alter immune responses, MSCs may offer a novel therapeutic to ameliorate the immune abnormalities apparent in some children with autism [13,14].

MSCs have profound immunoregulatory properties and are currently being investigated as a novel cellular immunomodulatory and anti-inflammatory agent in numerous clinical trials [30]. It has been shown that MSCs can reduce the proliferative capacity of T cells, B cells, NK cells, DC and neutrophils, and modulate a variety of immune cell functions: cytokine secretion and cytotoxicity of T cells and NK cells, B cell maturation and antibody secretion, DC maturation and activation, as well as antigen presentation [13,31]. In addition, MSCs can secrete a plethora of growth factors, anti-inflammatory cytokines and immunomodulatory mediators, such as indoleamine 2,3-dioxygenase, prostaglandin E2 (PGE2), nitric oxide, histocompatibility leucocyte antigen-G, transforming growth factor-β, interferon-γ, hepatocyte growth factor (HGF), IL-6, IL-10 and heme oxygenase-1 [14,32]. Recently, the mechanisms and molecules involved in the immunoregulatory effect of UCMSCs have been broadly revealed: suppressing the proliferation of B cells by modifying the phosphorylation pattern of Akt and p38 pathways [33]; mediating suppressive effects on T cell proliferation through monocytes as an essential intermediary [34]; and exerting immunomodulatory effects by PGE2-mediated mechanism [35]. The administration of UCMSCs to treat systemic lupus erythematosus has provided additional evidence for their immunoregulatory role [16,36], supporting their use in controlling both autoimmunity and triggered inflammation.

Several studies have corroborated that cerebral hypoperfusion is associated with many core symptoms in autism [37-41]. Generalized brain hypoperfusion, peaking in frontal and prefrontal regions, was observed in children with autism and associated with cognitive and neuropsychological defects [38]. In addition, decreased cerebral perfusion, especially in the temporo-parietal areas, has been related to cognitive impairment, such as language deficits, impairment of cognitive development and object representation, and abnormal perception and responses to sensory stimuli [39]. Inadequate perfusion resulting in brain tissue hypoxia not only caused neuronal apoptosis and necrosis, but also led to abnormal brain tissue metabolism and accumulation of pathological levels of neurotransmitter [40]. Therapeutically targeting cerebral ischemia and resulting hypoxia may be an alternative therapeutic approach in autism [41]. Therapeutic angiogenesis promoted by systemic administration of cord blood CD34^+^ stem cells to overcome ischemia has been experimentally demonstrated *in vitro* and animal models. It has been proved that the endothelial progenitor cell, contained in a CD34^+^ cell population enriched in CBMNCs, has the capacity to trigger angiogenesis in the ischemic tissues [42]. The circulating CD34^+^ progenitors in CBMNCs with the potential for endothelial development were recruited to the injury sites and developed into new endothelial cells to either repair the injured endothelial wall or sprout new vascular structure [43]. Moreover, human CD34^+^ cells and hematopoietic precursors can secrete numerous angiogenic factors, such as vascular endothelial growth factor (VEGF), HGF, and insulin-like growth factor-1 [44]. These promising results with CBMNC therapies have been successfully translated into the pre-clinical application for functional recovery in various ischemic animal models through the enhancement of angiogenesis around the site of degeneration [9,45]. Given the potency of cord blood CD34^+^ cells to promote angiogenesis in ischemic areas, the CBMNC may be useful for the improvement of the cerebral hypoperfusion and hypoxia that has been suggested to occur in the brains of individuals with autism [37-41].

In this present study, we compared the therapeutic efficacy of three groups: CBMNC transplantation with rehabilitation therapy, combined transplantation of CBMNCs and UCMSCs with rehabilitation therapy, and rehabilitation therapy alone. The data demonstrated that stem cell transplantation was more efficacious than conventional rehabilitation therapy in improving some features of autism. The mechanisms involved in improving the autistic symptoms might be through increased perfusion in brain areas and/or the regulation of immune dysfunction. Moreover, the Combination group showed overall more robust therapeutic efficacy than the CBMNC group, which may be attributed to the action of CBMNCs and UCMSCs in synergy that exert additional therapeutic benefits. In addition to immunoregulation, the cascade of cytokine spectrum triggered by UCMSCs is supportive of hematopoiesis: promoting the homing and expansion of transplanted CD34^+^ hematopoietic stem/progenitor cells to boost engraftment, such as stromal-derived factor-1; associating with hematopoietic stem cell proliferation, for example stem cell factor, macrophage colony-stimulating factor, granulocyte macrophage colony-stimulating factor and granulocyte colony-stimulating factor; and enhancing angiogenesis and tissue repair by VEGF [46]. Transplanted MSCs might integrate into the altered brain and restore damaged functions, promote synaptic plasticity and functional recovery, and rescue cerebellar Purkinjie cells in autistic subjects [47]. However, the exact mechanisms of CBMNC and UCMSC transplantation to treat autism still remain unconfirmed and need to be further clarified.

CARS, CGI and ABC scales were adopted to assess the therapeutic efficacy in this study. CARS can provide descriptive information about the pathological behavior and classify the degree of severity in autistic children, while the CGI scale, as a global measure, indicates the noticeable overall effect of treatment. However, further follow up studies will need to expand the behavioral assessments to include the more standardized measures in the Autism Diagnostic Observation Schedule. Studies have demonstrated that ABC is suitable for clinical quantitative evaluation of specific symptoms in children with autism [19,48]. Recently, the reliability and validity of the ABC Chinese Version (accurately translated from the original English version) for the measurement of therapeutic efficacy in Chinese children with autism have been examined, which showed a high positive correlation (r = 0.27 ~ 0.67, p < 0.01) with the CARS, Autism Behavior Checklist, Conner Parent Symptom Questionnaire and Achenbach Child Behavior Check list [49]. A close correlation between the ABC and CARS assessment results was consistently found in this study at each evaluation point increasing sequentially from baseline to 24w, also suggesting that the ABC is a useful measure for the evaluation of therapeutic efficacy in Chinese children with autism.

There are several limitations to this proof-of-concept study. First, the subjects were not randomized and they were not prospectively stratified based on disease severity or other demographic variable. Fortunately, there was no significant difference in the enrolled participants’ diagnostic, cognitive, or physical health among three groups, which allows for a valid comparison between groups. Second, the number of the subjects enrolled in this study was comparatively small, which may introduce bias in the safety and efficacy measures. Third, the subjects were followed for only 24 weeks and the long-term safety and efficacy were not evaluated. Fourth, neither the subjects nor evaluators were blinded, again possibly introducing bias into the measurements. Finally, the exact action mechanism was not known or clarified in this study.

## Conclusion

In summary, CBMNC and UCMSC transplantation may improve some behavioral symptoms and function observed in children with autism. This study presents for the first time a safety and efficacy analysis of using allogeneic CBMNCs and UCMSCs to treat behaviors in addition to conventional behavioral therapy in autism. With the safety profile of stem cell transplantation and the efficacy documented in this proof-of-concept study, large-scale randomized controlled double-blind studies are warranted to better define this novel therapeutic intervention on the long-term safety and efficacy in treating autism.

## Competing interests

XH is a shareholder of Shenzhen Beike Cell Engineering Research Institute. YZ, JQ and ZW are employees of Shenzhen Beike Cell Engineering Research Institute. No other authors declare any competing interests.

## Authors’ contributions

YL conceived of the study, participated in its design and coordination, carried out the clinical treatment and helped to draft the manuscript. YZ analyzed and interpreted data and drafted the manuscript. ML, YH, RG, XC carried out clinical treatment, evaluation of treatment efficacy, follow-ups and data collection. JQ and ZW carried out cell preparation, data collection, and statistical analysis. PA, SCC, BJK analyzed and interpreted data and helped to draft the manuscript. XH conceived of the study, participated in its design and coordination and helped to draft the manuscript. All authors read and approved the final manuscript.

## Supplementary Material

Additional file 1: Table S1 CARS Scores. **Table S2.** Correlation of ABC and CARS Scores. Click here for file
