# Supplementary material for: Transplantation of human cord blood mononuclear cells and umbilical cord-derived mesenchymal stem cells in autism
Source: J Transl Med. 2013 Aug 27;11:196. doi: 10.1186/1479-5876-11-196 (PMC3765833; doi:10.1186/1479-5876-11-196)
Supplement: Additional file 1: Table S1 — CARS Scores. Table S2. Correlation of ABC and CARS Scores. [file 1479-5876-11-196-S1.docx]

**Additional files**

**Additional table 1. CARS Scores**

| CARS item | Group | Baseline | 4w | | 8w | | 16w | | 24w | |
| --- | --- | --- | --- | --- | --- | --- | --- | --- | --- | --- |
| Relating to people | CBMNC | 3.5±0.76 | ^*^3.0±0.88 | ^*^2.93±0.83 | | ^*^2.86±0.86 | | ^*^2.79±0.89 | |  |
|  | Combination | 3.11±0.78 | 2.89±1.05 | 2.89±0.6 | | 2.44±0.53 | | 2.22±0.44^ab^ | |  |
|  | Control | 3.54±0.52 | 3.46±0.52 | 3.38±0.51 | | 3.15±0.55 | | ^*^3.0±0.41 | |  |
| Imitation | CBMNC | 3.36±0.74 | 3.0±0.78 | 2.57±0.94 | | ^*^2.57±0.85^a^ | | ^*^2.50±0.76^a^ | |  |
|  | Combination | 3.22±0.83 | 2.89±0.93 | 2.67±0.71 | | ^*^2.22±0.67^a^ | | ^*^1.78±0.44^a^ | |  |
|  | Control | 3.38±0.77 | 3.23±0.73 | 3.15±0.69 | | 3.15±0.69 | | ^*^2.77±0.44 | |  |
| Emotional response | CBMNC | 3.14±0.77 | 2.71±0.73 | ^*^2.07±0.62 | | 2.57±0.76 | | 2.57±0.76 | |  |
|  | Combination | 3.0±0.87 | 2.89±0.78 | 2.44±0.73 | | 2.11±0.6^a^ | | 2.11±0.6^a^ | |  |
|  | Control | 3.0±0.58 | 3.0±0.58 | 2.31±0.48 | | 3.08±0.64 | | 2.85±0.55 | |  |
| Body use | CBMNC | 2.79±0.8 | 2.21±0.58 | 2.07±0.62 | | 2.5±0.65 | | 2.29±0.47 | |  |
|  | Combination | 2.89±0.93 | 2.22±0.97 | 2.44±0.73 | | 2.0±0.71 | | ^*^1.67±0.5^ab^ | |  |
|  | Control | 2.46±0.52 | 2.46±0.52 | 2.31±0.48 | | 2.23±0.44 | | 2.15±0.38 | |  |
| Object use | CBMNC | 2.79±0.89 | 2.43±0.85 | 2.43±0.76 | | 2.43±0.76 | | 2.29±0.73 | |  |
|  | Combination | 3.0±0.71 | 2.56±0.73 | 2.33±0.5 | | 2.0±0.71 | | ^*^1.56±0.53^b^ | |  |
|  | Control | 2.46±0.52 | 2.31±0.48 | 2.31±0.48 | | 2.31±0.48 | | ^*^1.92±0.28 | |  |
| Adaption to change | CBMNC | 2.5±0.85 | 2.43±0.85 | 2.14±0.95 | | 2.07±0.92 | | 1.86±0.77 | |  |
|  | Combination | 3.11±0.93 | 2.33±0.87 | 2.22±0.44 | | 1.78±0.44 | | ^*^1.56±0.53 | |  |
|  | Control | 2.46±0.66 | 2.31±0.63 | 2.31±0.63 | | 2.15±0.55 | | 2.0±0.41 | |  |
| Visual response | CBMNC | 3.29±0.83 | ^*^2.57±0.65 | ^*^2.29±0.83^a^ | | 2.36±1.01 | | 2.36±1.01 | |  |
|  | Combination | 3.22±0.67 | 2.44±0.88 | ^*^2.11±0.78^a^ | | ^*^1.78±0.67^a^ | | ^*^1.67±0.5^ab^ | |  |
|  | Control | 3.23±0.44 | 2.92±0.49 | 2.92±0.49 | | 2.77±0.6 | | 2.54±0.66 | |  |
| Listening response | CBMNC | 2.57±0.94 | 2.57±0.94 | 2.21±0.8 | | 2.14±0.66 | | 2.14±0.66 | |  |
|  | Combination | 2.89±0.78 | 2.22±0.67 | 2.0±0.71 | | 1.67±0.71 | | 1.67±0.71 | |  |
|  | Control | 2.38±0.51 | 2.31±0.48 | 2.23±0.44 | | 2.31±0.48 | | 2.15±0.38 | |  |
| Taste, smell, and touch response and use | CBMNC | 2.36±1.08 | 2.36±1.08 | 2.14±01.03 | | 2.21±0.97 | | 2.29±0.91 | |  |
|  | Combination | 2.67±1.0 | 1.89±0.78 | 1.78±0.83 | | 1.78±0.67 | | 1.44±0.53^ab^ | |  |
|  | Control | 2.15±0.38 | 2.15±0.38 | 2.15±0.38 | | 2.15±0.38 | | 2.08±0.28 | |  |
| Fear or nervousness | CBMNC | 2.36±0.93 | 2.0±0.88 | 2.0±0.88 | | 2.21±1.12 | | 2.0±0.96 | |  |
|  | Combination | 2.89±0.78 | 2.22±0.44 | ^*^1.56±0.53^a^ | | ^*^1.56±0.53 | | ^*^1.33±0.5 | |  |
|  | Control | 2.46±0.52 | 2.31±0.63 | 2.31±0.48 | | 2.15±0.38 | | 2.0±0.58 | |  |
| Verbal communication | CBMNC | 3.71±0.47 | 3.29±0.83 | 3.36±0.84 | | ^*^3.07±0.83 | | 3.14±0.86 | |  |
|  | Combination | 3.33±0.87 | 3.11±0.6 | 2.89±0.78 | | 2.67±0.71 | | ^*^2.33±0.71 | |  |
|  | Control | 3.46±0.52 | 3.38±0.51 | 3.23±0.73 | | 3.08±0.64 | | ^*^2.92±0.64 | |  |
| Nonverbal communication | CBMNC | 3.07±0.73 | 2.64±0.74 | 2.57±0.76 | | 2.71±0.99 | | 2.64±0.93 | |  |
|  | Combination | 3.11±0.78 | ^*^2.33±0.87 | ^*^2.11±0.6 | | ^*^2.11±0.78 | | ^*^2.0±0.87 | |  |
|  | Control | 2.54±0.66 | 2.62±0.65 | 2.62±0.65 | | 2.62±0.65 | | 2.31±0.48 | |  |
| Activity level | CBMNC | 3.21±0.7 | 2.71±0.83 | 2.5±1.02 | | 2.57±0.94 | | ^*^2.29±0.73^a^ | |  |
|  | Combination | 3.33±0.87 | ^*^2.67±1.0 | 2.44±0.53 | | ^*^2.0±0.5 | | ^*^1.67±0.5^a^ | |  |
|  | Control | 2.46±0.52 | 2.54±0.66 | 2.69±0.48 | | 2.54±0.52 | | 2.31±0.48 | |  |
| Intellectual response | CBMNC | 3.86±0.36 | 3.5±0.76 | 3.36±0.84 | | ^*^3.07±0.83 | | ^*^3.07±0.83 | |  |
|  | Combination | 3.44±0.88 | 3.33±0.5 | 3.11±0.6 | | 2.78±0.83 | | 2.67±0.71 | |  |
|  | Control | 3.23±0.44 | 3.23±0.44 | 3.31±0.48 | | 3.31±0.48 | | 3.15±0.38 | |  |
| General impressions | CBMNC | 3.21±0.58 | 3.07±0.62 | 2.93±0.62 | | 3.07±0.73 | | 2.93±0.83 | |  |
|  | Combination | 3.0±0.87 | 2.67±0.71 | 2.78±0.83 | | 2.56±0.53^a^ | | 2.33±0.5^ab^ | |  |
|  | Control | 3.23±0.6 | 3.31±0.48 | 3.31±0.48 | | 3.31±0.48 | | 3.08±0.28 | |  |
| Total score | CBMNC | 46.43±8.65 | ^*^39.21±8.63 | ^*^36.64±7.07 | | ^*^35.14±7.77 | | 37.14±10.15 | |  |
|  | Combination | 45.11±4.31 | 40.67±3.82 | 38.22±9.74 | | 36.78±12.8 | | ^*^28.00±6.18^ab^ | |  |
|  | Control | 43.15±4.38 | 41.54±3.82 | 41.46±3.41 | | 40.31±3.82 | | ^*^37.23±3.42 | |  |

**Additional table 2. Correlation of ABC and CARS Scores**

|  | Baseline | 4w | 8w | 16w | 24w |
| --- | --- | --- | --- | --- | --- |
| CARS | 44.91±6.38 | 40.41±6.99 | 38.77±6.98 | 37.41±8.38 | 34.88±8.20 |
| ABC | 86.77±21.40 | 74.08±19.14 | 66.00±22.11 | 61.27±24.29 | 54.38±24.34 |
| *R* | 0.6064 | 0.6728 | 0.7452 | 0.8131 | 0.8707 |
| *P* | <0.001 | <0.001 | <0.001 | <0.001 | <0.001 |

Note: CARS denotes Childhood Autism Rating Scale, ABC Aberrant Behavior Checklist, and *r* [Pearson correlation coefficient](http://en.wikipedia.org/wiki/Pearson_product-moment_correlation_coefficient).
